# Degranulation of human mast cells: modulation by P2 receptors’ agonists

**DOI:** 10.3389/fimmu.2023.1216580

**Published:** 2023-10-05

**Authors:** Edward S. Schulman, Haruhisa Nishi, Amir Pelleg

**Affiliations:** ^1^Division of Pulmonary, Critical Care and Allergy, Drexel University College of Medicine, Philadelphia, PA, United States; ^2^Department of Pharmacology, Jikei University School of Medicine, Tokyo, Japan; ^3^Danmir Therapeutics, LLC, Haverford, PA, United States

**Keywords:** human lung mast cells, LAD2 cell line, P2 purinergic receptors, allergic degranulation, FcϵRI, PI3K(δ), P2Y11R

## Abstract

Since the late 1970s, there has been an alarming increase in the incidence of asthma and its morbidity and mortality. Acute obstruction and inflammation of allergic asthmatic airways are frequently caused by inhalation of exogenous substances such as allergens cross-linking IgE receptors expressed on the surface of the human lung mast cells (HLMC). The degree of constriction of human airways produced by identical amounts of inhaled allergens may vary from day to day and even hour to hour. Endogenous factors in the human mast cell (HMC)’s microenvironment during allergen exposure may markedly modulate the degranulation response. An increase in allergic responsiveness may significantly enhance bronchoconstriction and breathlessness. This review focuses on the role that the ubiquitous endogenous purine nucleotide, extracellular adenosine 5’-triphosphate (ATP), which is a component of the damage-associated molecular patterns, plays in mast cells’ physiology. ATP activates P2 purinergic cell-surface receptors (P2R) to trigger signaling cascades resulting in heightened inflammatory responses. ATP is the most potent enhancer of IgE-mediated HLMC degranulation described to date. Current knowledge of ATP as it relates to targeted receptor(s) on HMC along with most recent studies exploring HMC post-receptor activation pathways are discussed. In addition, the reviewed studies may explain why brief, minimal exposures to allergens (e.g., dust, cat, mouse, and grass) can unpredictably lead to intense clinical reactions. Furthermore, potential therapeutic approaches targeting ATP-related enhancement of allergic reactions are presented.

## Introduction

Within minutes of allergic activation of IgE receptors expressed by HMC, the cells release histamine and a spectrum of other pro-inflammatory mediators to induce airway bronchoconstriction and contribute to acute and chronic lung inflammation (1–3). Preformed cytoplasmic granular mediators are released over seconds (e.g., histamine and TNF alpha), select chemicals are newly formed over minutes (e.g., leukotrienes and other lipids), and other mediators including cytokines over hours (e.g., Interleukin (IL) -5, IL-13, IL-8, and GM-CSF) (1, 4). Depending on the strength of the stimulation, the localized inflammatory milieu, and the organs involved, mast cell (MC) activation can result in multiple responses including edema, hives, bronchoconstriction, or systemic anaphylaxis, which can not only decrease the quality of life but may also be life-threatening. Many publications have described studies of “human mast cells.” Most of these studies have used neoplastic MC (5–7) or cells cultured *in vitro* derived from differing precursor cells (e.g., cord blood, peripheral blood, and fetal liver), using culture media that include various combinations of cytokines (8–19). Relatively few studies used freshly isolated and purified human lung mast cells (HLMC) as a model of the organ-specific responses of the human lung (20). Also, there is increasing appreciation that MC are heterogeneous, and their biology can differ markedly not only between species (e.g., mouse vs. human) but also between MC isolated from different human organ sources (e.g., skin vs. lung), within the same organ (e.g., gut), and freshly isolated organ-derived MC vs. *in vitro*-derived MC (8, 21–26).

Progress in understanding HLMC biology has been extremely slow because of difficulties in procuring freshly resected human specimens, time-consuming and technical challenges associated with the isolation and purification of these cells, and their limited survival *in vitro* (i.e., 2-4 days). Our seminal report on the methods of isolation and purification of HLMC (27) facilitated many subsequent studies on their biology including ultrastructure (28–32), heterogeneity (33–35), mediators’ release biochemistry (36, 37), secretagogues (38–45), mediators (46–55), and pharmacological modulation of mediators’ release (40–42, 45, 56).

The text below focuses on the progress made in recent years in our understanding of the critical interaction of the extracellular purine nucleotide adenosine 5′-triphosphate (ATP), with IgE-mediated activation of the HLMC (57, 58) and intracellular signal transduction pathway associated with IgE receptor activation in LAD2 cell line (6, 59, 60). Our own interest in this regard mostly relates to allergic asthma, but other important aspects of ATP’s role in pulmonary pathophysiology have been investigated including cystic fibrosis, pulmonary embolism, cough, bronchoconstriction, pulmonary fibrosis, lung cancer, mechanical ventilation-induced lung injury, and pulmonary hypertension (61–68).

### Intracellular ATP

Intracellular ATP plays a critical role in cellular metabolism and energetics (69). ATP is found at a concentration of 5–10 mM in every cell, except for platelets, in which its concentration is far higher. The concentration of ATP in chromaffin cells’ secretory granules approaches 100 mM; platelets’ ATP level is up to 500 mM. However, the concentration of extracellular ATP is only approximately 10 nM (70)(see below).

### Extracellular ATP

#### Release of ATP from cells

In 1929, Drury and Szent-Gyorgyi described the effects of a simple extract of heart muscle and other tissues on the cardiovascular system. The active ingredient in this extract was identified as adenylic acid (71). Subsequent studies have shown that adenosine and ATP were the most active vasodilators and bradycardic ingredients of these extracts (72–74). ATP is released from cells by various mechanisms under physiologic and pathophysiologic conditions in response to different stimuli or micro-environmental conditions. These include exocytosis, large membrane pores, and specific trans-cell membrane ionic channels (75, 76). There are several sources of extracellular ATP (23, 77). Large amounts of ATP are found in platelets and ATP is released during platelet activation. Upon platelet aggregation, the serum concentration of ATP and adenosine diphosphate (ADP) reaches 50 uM but is much higher at the cell surface (78–80). ATP is also stored in red blood cells (RBC) from which it is released under conditions of imbalance between O_2_ supply and O_2_ demand (81–84). In addition, several biologic substances as well as increased blood flow can induce the release of ATP from vascular endothelial cells (77, 85–88) and smooth muscle cells (89, 90). Other ATPs release stimuli and cellular sources including mechanical deformation of cells, ischemic cells, immune cells, and necrotic/apoptotic cells (23, 91–94). Pannexin channel and connexin hemichannel play a critical role in this release (95). ATP is also released from neural elements as a co-transmitter and from exercising skeletal muscles (96). In the heart, ATP is released into the extracellular fluid under various conditions. Specifically, ATP release is evoked by sympathetic nerve stimulation and by catecholamines (97–101). In addition, ATP is released in the heart during acute myocardial ischemia (102) and from cardiac myocytes in response to hypoxia (103, 104).

Importantly, during inflammation, ATP is released from inflammatory cells. For example, elevated extracellular levels of ATP have been found in the lungs of COPD patients (105, 106). Similarly, pulmonary levels of ATP were increased in a mouse model of smoke-induced acute lung inflammation and emphysema (107–109).

#### Degradation of extracellular ATP

Extracellular ATP is rapidly and sequentially degraded by ectonucleotidases including ectonucleoside triphosphate diphosphorylase-1 (CD39), and ecto-5’-nucleotidase (CD73). CD39 hydrolyzes extracellular ATP and ADP to AMP. CD73 catalyzes the hydrolysis of AMP, releasing inorganic phosphate and adenosine, which exerts its own effects by activating P1 purinergic receptors (A1, A2a, A2b, and A3) (110–113).

CD73 is widely expressed in a variety of tissues, including the colon, kidney, brain, liver, heart, lung, spleen, and bone marrow (114). CD39 is expressed by multiple cell types including epithelial, endothelial, and immune cells. It is highly expressed in different human tumor types (115). Adenosine is rapidly eliminated from the extracellular space by ectoadenosine deaminase and actively transported into cells (109). Therefore, the levels of CD39 and CD73 and their enzymatic activities play a critical role in controlling the duration and magnitude of autocrine and paracrine effects of ATP and adenosine. Multiple studies have shown that the level of these enzymes is altered during pathophysiologic conditions. For example, increased expression of CD39 and CD73 by pulmonary epithelial and endothelial cells was observed during high inspiratory pressure level-induced lung injury (116). Also, upregulation of CD39/CD73 expression has been observed in patients with small-cell lung cancer and patients with a broad spectrum of solid cancers (117).

#### ATP: A paracrine and autocrine agent

**Figure 1:**
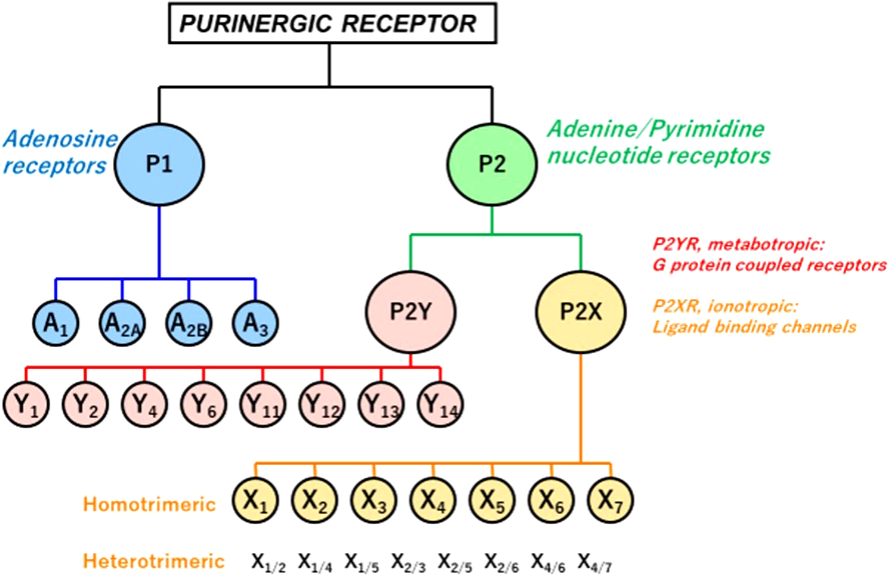
P1 and P2 Purinergic Receptors. Extracellular signals triggered by ATP, its hydrolytic products ADP and adenosine, and other nucleotides and nucleotide sugars (UTP, UDP, and UDP-glucose) regulate physiological and pathophysiological processes. Two families of purinoceptors are shown. P1R consists of four subtypes of adenosine-activated receptors. P2R is divided into P2XR ionotropic ligand-gated ion channel receptors, principally activated by ATP, P2YR, and G-protein-coupled (metabotropic) receptors. Also shown are multiple heteromeric assemblies comprising P2X subunits, but not all have been detected in native tissues (119).

Extracellular ATP acts as a paracrine and autocrine agent (23, 91, 118), the actions of which are mediated by cell surface purinergic receptors (P2R) (109). The latter are divided into two families: P2Y: seven trans-cell membrane domain G-protein coupled receptors (metabotropic), and P2X: trans-cell membrane cationic channels (ionotropic). Eight P2YR and seven homotrimeric P2X receptors (P2X1-7) have been cloned heretofore (**Figure 1**). Multiple heteromeric assemblies comprising P2X subunits have been described including P2X2/P2X3, P2X4/P2X6, P2X2/P2X6, and P2X1/P2X5, but not all have been detected in native tissues (119).

In contrast to P2XR, which is principally activated by ATP, P2YR shows striking differential sensitivity for varying nucleotides. Single cells commonly express more than one P2YR, for which a given nucleotide has an affinity. Pharmacologically, P2YR are subdivided into adenine-nucleotides sensitive group responding mainly to adenosine diphosphate (P2Y1R, P2Y12R, and P2Y13R: ADP), adenosine triphosphate (P2Y11R: ATP), uridine triphosphate (P2Y4R: UTP), uridine diphosphate (P2Y6R: UDP), UDP and sugar derivatives sensitive receptor (P2Y14R: UDP-glucose and UDP-galactose), and receptors manifesting mixed agonist affinity (P2Y2R: ATP=UTP) (120) (**Table 1**). ATP is also an antagonist or partial agonist at the P2Y1 receptor (121). Furthermore, whereas ATP activates human P2Y11 receptors, ADP is a canine P2Y11 receptor agonist (122). P2Y1, -2, -4, -6, and -11 receptors belong to the P2Y1-like subfamily and couple to G_q/11_, G_o_, G_12/13_, and G_s_ protein, whereas P2Y12, -13, and -14 receptors are categorized as P2Y12-like and couple to G_i/o_ protein. ATP is the only agonist that can activate both P2XR and P2YR (23, 123) (**Table 1**).

**Table 1:** P2Y Receptor Family.

RECEPTOR	PREFERRED AGONIST	G-PROTEIN COUPLING	COMMENTS
P2Y1	ADP>>ATP	G_q_	ATP can be an agonist, depending on receptor reserve (antagonist or partial agonist).
P2Y2	ATP=UTP	G_q_ (+ G_i_)	No agonist activity of ADP
P2Y4	UTP	G_q_ (+ G_i_)	ATP is a partial agonist in humans and a full agonist in rat
P2Y6	UDP	G_q_	Uracil nucleotides play a role as intercellular messengers independent of adenine nucleotides.
P2Y11	ATP	G_q_ + G_s_	No murine P2Y11 gene
P2Y12	ADP	G_i_	
P2Y13	ADP	G_i_	
0P2Y14	UDP-Glucose UDP-Galactose	G_i_	

Inhalation of aerosolized ATP triggered bronchoconstriction in healthy and more so in asthmatic human subjects; in the latter, ATP was 50-fold more potent than methacholine and 87-fold more potent than histamine in producing a 15% decrease in FEV1 (124). In 2002, based on multiple studies in both canine and human models (124–126), a mechanistic role of ATP in pulmonary disorders was proposed for the first time in a seminal review, and termed “*Adenosine 5′-triphosphate axis in obstructive airway diseases*” (127, 128).

### ATP enhances IgE-mediated HLMC degranulation

Early experiments in HLMC suggested that ATP plays an important modulatory role in degranulation as measured by the percentage of total cellular histamine released within minutes following IgE-mediated challenge (58). In these experiments, freshly purified HLMC (10-50 x 10^3^/tube) were incubated with or without purine nucleotides for 15 min prior to a 20 min challenge with anti-IgE (3 ug/ml), calcium ionophore A23187 (0.1 ug/ml), or buffer control. Cells were exposed to ATP, UTP, the stable ATP analogs α, β methylene-ATP (α, β-MeATP), (β, γ methylene-ATP (β, γ-MeATP), and 2-methylthio-ATP (2-MeSATP), as well as adenosine, the product of ATP’s degradation.

Neither the nucleotides (10^-7^-10^-3^ M) nor adenosine (10^−6^–10^−3^ M) directly triggered degranulation, contrary to results obtained using murine mast cell models (129–133). In HLMC, adenosine exhibited a bimodal effect on anti-IgE-induced histamine release, enhancing it at 10^−6^ to 10^−4^ M (P > 0.05, NS) and inhibiting it at 10^−3^ M (P < 0.05) (58), in congruence with prior reports on HLMC (41, 134, 135). ATP (10^−4^ M) enhanced anti-IgE–induced histamine release (10.9 ± 2.7% to 19.2 ± 2.9%, n = 20, P < 0.01). Importantly, ATP’s effects were most striking in cells manifesting low (< 3%) IgE-mediated net histamine release termed a weak allergic stimulation (WAS). When WAS-dependent histamine release was “low”, i.e., 1.8 ± 0.4%; range: 0.5–2.9%; n=6), ATP (10^-4^ M) enhanced histamine release to 13.5 ± 2.7% (750% enhancement). In contrast, ATP had no effects on the ionophore A23187-induced histamine release (n = 10). All adenine nucleotides enhanced IgE-mediated HLMC histamine release, but ATP was the most potent followed by 2-MeSATP, α, β-MeATP, and β, γ-MeATP. This potency order strongly suggests that the action of ATP is mediated by a P2YR subtype (136). Further evidence against the involvement of a P2XR, especially P2X7R, was the failure of the selective P2XR antagonist pyridoxalphosphate-6-azophenyl-2′,4′-disulfonic acid (PPADS) (137) to affect ATP’s enhancement.

The P2YR- agonist UTP also significantly enhanced anti-IgE–induced histamine release, which is most consistent with a P2YR and not P2XR mediation. However, UTP was less potent than ATP. Control anti-IgE–induced histamine release of 14.9 ± 3.9% was enhanced by ATP (10^-4^ M) to 23.0 ± 4.7% (p < 0.05), compared with 19.2 ± 5.0% (p < 0.05) in the presence of an equimolar concentration of UTP. This result did not support sole mediation by P2Y2R, of which UTP is the preferred agonist over ATP (138).

### HLMCs fail to exhibit functional ecto-ATPase activities

Over the course of 15-minute incubations with ATP, HLMC’s media did not contain breakdown products of ATP, indicating that the histamine-release enhancement by ATP was not mediated by ADP, AMP, or adenosine. It should be noted that under identical experimental conditions, products of ATP’s degradation were found in media containing whole fragments of the human lung (58).

### Expression of P2Rs in HLMC

Further support for a P2YR mediating the effects of ATP on IgE-mediated degranulation enhancement from HLMC was the constitutive expression of P2Y1R and P2Y2R and weak expression of P2Y4 demonstrated by reverse transcription–polymerase chain reaction (RT-PCR) of freshly purified HLMC (58). A receptor, previously known as P2Y7, was weakly manifested in the RT-PCR assay in one of five individual fresh isolations of HLMC. P2Y7 was later identified as the receptor for leukotriene B4 (B-LTR) (139). Feng et al. reported the expression of P2Y1, P2Y2, P2Y11, P2Y12, and P2Y13 by RT-PCR in cord blood-derived human MC and highlighted the potential roles of different P2YR in complementary or opposing functions in cell activation (140). Wareham et al. reported P2X7 expression by RT-PCR in HLMC that were cultured in a cytokine mix pre-experimentation. In addition, using a whole-cell patch-clamp technique, they described a P2X7-like non-desensitizing current in response to high concentrations of ATP (1–5 mM). P2X1 and P2X4 transcripts were also found in their HLMC preparations (141).

Stimulation of the P2YRs can result in the activation of two different pathways: one that involves pertussis toxin (PTX)-sensitive G protein(s) and adenylyl cyclase (G_i_-coupled; P2Y12, P2Y13, and P2Y14), and the other that involves PTX-insensitive G protein and phospholipase C (G_q_-coupled; P2Y1, P2Y2, P2Y4, P2Y6, and G_q/s_-coupled; P2Y11) (142). Because PTX failed to modify the enhancing effect of ATP on IgE-mediated histamine release, it seems likely that the latter pathway mediates the effect of ATP (58). However, the exact post-P2YR mechanism(s) by which this pathway(s) operates to enhance IgE-mediated degranulation in HLMC remains to be elucidated.

### ATP enhances IgE-mediated LAD2 HMC degranulation

Though P2YRs are expressed by HMC of different sources (58, 140, 143), until recently, only one receptor (i.e., P2Y14R) in LAD2 cells was proposed to be linked to allergen/IgE-induced degranulation (60). To further elucidate the specific receptor(s) and, importantly, post-receptor pathways accountable for nucleotide-P2YR mediated effects on IgE-mediated degranulation, sufficient cell numbers were needed beyond what the HLMC model could provide. The LAD2 human-derived MC line was developed at the NIH from a patient with systemic mastocytosis (5, 6, 10). As recently reported, LAD2 cells express five P2YRs (P2Y1, P2Y6, P2Y11, P2Y12, and P2Y14) along with three P1Rs (A2aR, A2bR, and A3R), and two P2XRs (P2X1 and P2X7) (59). LAD2 cells express CD39 but not CD73, suggesting extracellular nucleotides could be degraded to AMP but not to adenosine (59).

In non-sensitized LAD2 cells challenged with P1R and P2R agonists [adenosine, ATP, ADP, UTP, UDP, 2-methylthio-ATP (2-MeSATP)], and the P2Y11R non-hydrolysable agonist [adenosine 5’-(3-thio) triphosphate (ATPγS)] at physiological concentrations (1–100 μM), none of the agonists alone triggered degranulation as measured by histamine or beta-hexosaminidase release. High concentrations (1mM) of BzATP, a P2X7R agonist, triggered degranulation in non-sensitized LAD2 cells. Sensitized LAD2 cells were then challenged in the absence and presence of P2YR agonists, using a predetermined low concentration of antigen to produce a weak allergic stimulation (WAS) and a low level of degranulation (<10% release). As noted above for HLMC, ATP’s enhancement was most pronounced under WAS conditions; only the P2Y11R non-hydrolyzable agonist, ATPγS (144–147), mimicked the effects of ATP and produced a net 7-fold increase in release (n = 4; p < 0.01). The failure of UTP and ADP to enhance the release of histamine excluded the involvement of their respective receptors, P2Y1R, P2Y2, P2Y4R, P2Y12R, and P2Y13R. P2Y11R protein expression by LAD2 cells was confirmed by Western blot. The effects of ATPγS were dose-dependently inhibited by NF157, a P2Y11R antagonist (148, 149). Further evidence for P2Y11 as the receptor mediating the degranulation-enhancing effect of ATP in LAD2 cells is its known coupling to both G_q/11_ and G_s_ proteins that are known to be linked to induction and suppression of MC degranulation, respectively.

**Figure 2:**
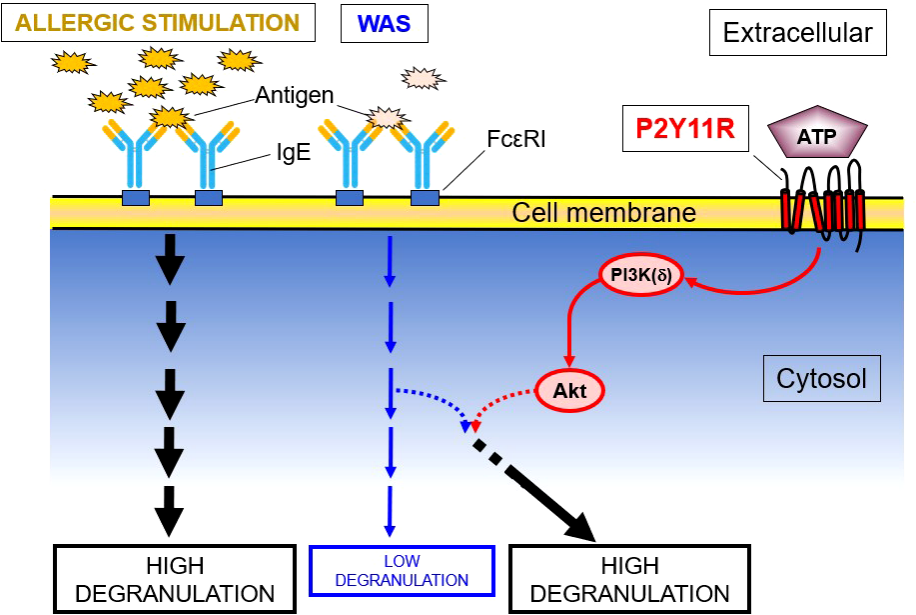
P2YR11 Modifies a WAS in LAD2 Human MC. This schematic shows that P2Y11R, an endogenous purine (ATP) receptor, modulates the enzymatic cascade between PI3K(δ) and Akt, leading to the enhancement of a weak allergic stimulus (WAS). PDK-1, though a known link between PI3K and Akt, is not phosphorylated and is not involved in the degranulation enhancement.

None of the P2YR agonists tested in LAD2 cells, including high concentrations of ATPγS (1000 μM) enhanced the WAS-induced intracellular Ca^2+^ mobilization, which is an essential component of activated FcϵRI-induced degranulation. The effects of ATPγS on the phosphorylation of key kinases related to intracellular PI3K(δ)’s activation cascades showed that both PI3K(δ) and Akt were phosphorylated by ATPγS and are further upregulated by WAS, especially in the case of Akt. However, PDK-1, which is known to be a link between PI3K(δ) and Akt, was not phosphorylated. These data indicated that the P2YR-mediated enhancement effect on IgE-mediated degranulation in an HMC is via the PI3K/protein kinase B pathway (**Figure 2**). In further experiments, a shRNA directed against PI3K(δ) and a PI3K(δ) inhibitor, compound 15e (150, 151), suppressed ATPγS’s effect on WAS-induced degranulation enhancement, the latter in a concentration-dependent manner. In additional experiments, an antagonist of the P2Y11R and NF157 significantly inhibited the enhancing effects of ATPγS (100 μM) on WAS-induced degranulation and experiments using siRNA knockdown of the P2Y11R, which itself abolished the enhancing effects (59).

## Discussion

### Historical perspective

Over the past four decades, adenosine, but not adenine nucleotides, has received considerable attention for a potential role in human allergies and asthma (41, 57, 130, 134, 135, 152–156). In 1996, Pelleg and Hurt showed for the first time that extracellular ATP stimulates vagal sensory nerve terminals in the canine lungs by activating P2XR (125). Subsequent studies have indicated that this action of ATP leads to bronchoconstriction and cough and is probably also pro-inflammatory due to the localized release of neuropeptides via the axon reflex (62, 157, 158). The suggestion that this latter pathway could be mechanistically involved in asthma (159) prompted us to investigate the potential effects of extracellular ATP on HLMC. Our early findings indicated that ATP potentiates IgE-mediated degranulation from HLMC (58). Subsequent studies have shown a similar phenomenon in LAD2 cells, which was not mediated by adenosine, which is the product of ATP’s degradation by ecto-enzymes. Since then, additional studies have investigated the role of extracellular and intra-MC ATP in MC’s role in allergic inflammation (160).

### Mechanisms of actions of ATP

#### Mediation by P2R

ATP is unimodal, only enhancing degranulation, whereas adenosine is bimodal; at high concentrations, it inhibits degranulation and at low concentrations, it potentiates degranulation. At equimolar concentrations, ATP is more potent than adenosine in enhancing HMC degranulation (57–59). ATP’s enhancing effects on IgE-mediated degranulation in freshly isolated HLMC, without exposure to varied cytokine mixtures over days to weeks in cultures, is substantial and reproducible. The specific P2R subtype (s) mediating this degranulation-enhancing effect in HLMC remain unknown, but several lines of evidence strongly suggest the involvement of P2YR and not P2XR. It is also possible that two or more P2YRs are activated in concert during different physiologic conditions. Along these lines, Feng et al. reported the expression of multiple P2YRs in human cord blood-derived MC, including P2Y1R, P2Y2R, P2Y11R, P2Y12R, and P2Y13R, that could play complementary and opposing functions during mast cell activation (140). Regrettably, the difficulties involved in procuring specimens of human lungs and isolating and purifying HLMC stymied efforts to fully elucidate the relevant mechanisms of ATP’s enhancement of the immune reaction-dependent MC degranulation.

More recently, insights into pathways involving P2YR expressed by HMC were obtained by quantifying the effects of ATP on LAD2 cells, which are proliferating neoplastic HMC lines. However, LAD2 cells are more analogous to MCTC-type HMC which predominate in skin and other organs and represent a distinct minority population in the human lung where MCT predominates (25, 161–163). That notwithstanding, LAD2 cells do show similarity to HLMC: ATP does not trigger degranulation of these cells by itself as it does in murine MCs (130, 132, 141, 164), and ATP-induced enhancement of IgE-mediated degranulation is observed in both HLMC and LAD2 cells.

There is no evidence for P2X7R expression in freshly isolated HLMC which contrasts with the positive RT-PCR for P2X7R and functional activity under whole-cell patch clamp conditions reported in cultured HLMC by Wareham et al. (141). The latter also reported that HLMC express P2X1R and P2X4R and suggested that the differential findings related to P2X7R could be the result of different cell isolation techniques and/or differences in HLMC receptor expression at the time of fresh isolation vs. the pre-experimentation culture of the cells in cytokine-containing mixtures. However, we have performed long-term HLMC culture (2 weeks to 4 months) in a medium containing a combination of Stem Cell Factor and IL-4 (56) and still failed to identify the expression of P2X7R using RT-PCR. By contrast, RT-PCR assays showed that the HMC-1 mast cell leukemia line (58) and the LAD2 cell line do robustly express P2X7R, and in the latter’s case, when challenged with BzATP triethylammonium salt, the prototypic P2X7R agonist will lead to degranulation. This agonist failed to trigger degranulation in HLMC (58).

Matsuoka et al. (165) and Yoshida et al. (166) clearly demonstrated that P2X4R activation potentiated Mas-related G protein-coupled receptor X2 (MrgprB2)-mediated pseudo-allergic responses in murine MC. MrgprB2 is the mouse ortholog of human MRGPRX2. However, few reports have demonstrated potentiating effects on P2X4R-stimulated allergic responses in HMC. Bonvini et al. reported that a Transient receptor potential cation channel, subfamily V, member 4 (TRPV4) agonist will cause ATP release from, but not contraction of, isolated airway smooth muscle. Smooth muscle contraction required co-incubation with HLMC. TRPV4-mediated ATP release by the airway smooth muscle-stimulated HLMC to release cysteinyl leukotrienes via a P2X4R-dependent mechanism which subsequently induced smooth muscle contractions in an IgE-independent fashion (167). In any case, these reports suggest that P2X4R, but not P2X7, expressed on MC may also be associated with pathological effects.

Additional evidence for a P2YR subtype(s) and not a P2XR subtype mediating the effects of ATP on HLMC goes beyond transcriptional data. Specifically, the pattern of pharmacologic responsiveness to stable analogs of ATP (i.e., ATP > 2-MeSATP > α, β -MeATP > β, γ-MeATP) is consistent with mediation by P2YR and not P2XR (23, 91, 118, 136). Second, we examined the putative P2XR-selective antagonist PPADS (137) on ATP’s effects on enhancing IgE-mediated HLMC degranulation. The failure of this agent to influence ATP-induced enhancement of degranulation along with the previously stated failure of BzATP triethylammonium salt to stimulate HLMC release further argues against the involvement of P2XR.

ATP-induced enhancement of IgE-mediated degranulation in LAD2 cells shows responses linked to P2Y11R activation. This conclusion was based on (i) the robust expression of P2Y11R, (ii) the lack of enhancing effect of ADP and UTP, which excluded P2Y1R, P2Y2R, P2Y4R, P2Y12R, and P2Y13R, (iii) strong enhancing effects of ATPγS, which acts as an agonist at the P2Y11R (145, 168, 169), (iv) the marked inhibition of ATPγS’ effects by NF157, which is a selective P2Y11R antagonist (148, 149), and (v) the fact that P2Y11R is coupled to both G_q/11_ and G_s_ proteins (118), the former mediating enhancement of allergen-induced degranulation.

Gao et al. (60) reported that P2Y11R is the most robustly expressed among P2YRs in LAD2 cells, and P2Y11R agonists, including ATPγS, do not directly trigger degranulation. Gao et al. also investigated the role of P2YR in ATP’s enhancement of allergic degranulation in LAD2 cells. They failed to find an effect of ATPγS. However, their test dose was at 10 μM, whereas ours was 100 μM. In addition, those investigators studied the effects on maximal but not minimal antigen-induced degranulation; they also proposed that multiple P2Rs including UDP-glucose-sensitive P2Y14R may play a role in this action of ATP.

#### Intracellular signal transduction pathways

P2Y11R activation in LAD2 cells leads to activation of intracellular pathways involving PI3K/Akt (**Figure 2**). As previously reported, the PI3K/Akt pathway can operate without the induction of intracellular Ca^2+^ mobilization (170, 171). It is well known that IgE receptor (FcϵRI) activation itself, leading to degranulation, is associated with intracellular Ca^2+^ mobilization (172). Intracellular Ca^2+^-independent steps in MC degranulation have previously been reported (173–176). This suggests that the potentiating effect of extracellular ATP on anti-IgE–induced degranulation is due to a complex interaction between the two relevant signal-transduction pathways, rather than merely an increase in Ca^2+^ influx induced by ATP.

That PI3Kδ plays a critical role in ATP enhancement is shown by a dose-dependent inhibition of the ATPγS’ enhancing effects by compound 15e, a PI3K inhibitor (150, 151), and marked diminution of enhancing effects in PI3Kδ knockdown LAD2 cells (59). PDK-1 is a key element of the PI3K/Akt pathway (177–179), but the lack of PDK-1 phosphorylation does not support its involvement in ATPγS’ enhancement of WAS in LAD2 cells.

#### Modulation by ecto-enzymes

The enhancement of WAS by ATPγS in LAD2 cells was unrelated to its rate of breakdown by ectonucleotidases. However, expressions of multiple purinergic receptors, and/or up- or down-regulation of ectonucleotidases *in vivo* may affect cell responses to extracellular nucleotides. Experiments showed that LAD2 cells’ exposures to ATPγS did not affect extracellular concentrations of ATP for at least 60 minutes. However, under experimental conditions, ATP could be released from LAD2 cells.

We have previously reported that the half-life of extracellular ATP/ADP (1 mM) was 14.88 min in another human cell line expressing ectonucleotidases CD39, CD73, and alkaline phosphatase (180). Studies have emphasized the role of ectonucleotidases in the magnitude of ATP’s effects in pulmonary and other organ disorders (68, 181). Tsai and Takeda (160) have recently shown in mice that E-NPP3, an activation marker induced in IgE-mediated reactions, hydrolyzes extracellular ATP on basophil and MC surfaces to prevent ATP-dependent excess activation. In the absence of E-NPP3, basophils and MC become overactivated and the mice experience severe chronic allergic inflammation (160). This work suggests a potential therapeutic role for ATP hydrolysis strategies to control MC-mediated allergic responses. It increasingly appears that a complex of physiological factors interacts to either maintain or degrade extracellular ATP *in vivo*, depending on localized physiological conditions such as pH.

### Concluding remarks

Extracellular ATP is a potent modulator of immune reaction-induced HLMC degranulation and thereby release of inflammatory mediators. Since extracellular ATP is rapidly degraded to ADP and adenosine by ecto-enzymes, and adenosine exerts its own effects on inflammatory cells, the variable levels of these enzymes under physiologic and pathophysiologic conditions are critical modulators of the effects of extracellular ATP and adenosine in this setting.

Although the exact mechanism of ATP’s effect on HLMC degranulation has not been delineated heretofore, the voluminous relevant data strongly suggest that extracellular ATP plays an important mechanistic role in HLMC allergic reactions. Thus, because of the exponential research work in this arena over the past two decades since the original hypothesis of the *“ATP axis in the Lungs”* was put forward (127), we are now on the cusp of developing novel therapeutic approaches for allergic disorders in general and asthma in particular. This view is supported by the recent developments in a parallel field of ATP-chronic cough, in which pre-clinical studies and clinical trials with P2X3R antagonists are being evaluated as novel anti-tussive agents (182).

The effects of ATP are most pronounced with a concurrent WAS. It is plausible that the cellular ATP environment may change over short periods (e.g., exercise, irritants, etc.), while antigen exposure may not vary and/or remain weak (e.g., dust, cat, mouse, and grass), yet a full-blown asthmatic exacerbation or urticarial episode may ensue. The identity of the receptor(s) mediating the effects of ATP on HMC and the ectoenzymes that hydrolyze ATP in different tissues must be further explored so that novel therapeutic approaches can be tested in the clinical setting.

## Author contributions

The potential role of purine nucleotides in the function of human lung MCs under physiologic and pathophysiologic conditions was originally conceived by AP and ES. The HLMC experiments were supervised by ES. HN conducted all LAD2 experiments. ES wrote the first draft of the manuscript, and it was edited by HN and AP. All authors have read the final draft of the manuscript and approved its submission for publication.
